# Seniors managing multiple medications: using mixed methods to view the home care safety lens

**DOI:** 10.1186/s12913-015-1193-5

**Published:** 2015-12-12

**Authors:** Ariella Lang, Marilyn Macdonald, Patricia Marck, Lynn Toon, Melissa Griffin, Tony Easty, Kimberly Fraser, Neil MacKinnon, Jonathan Mitchell, Eddy Lang, Sharon Goodwin

**Affiliations:** Victorian Order of Nurses (VON Canada), Ottawa, ON Canada; Dalhousie University, Halifax, Nova, Scotia Canada; University of Victoria, Victoria, BC Canada; University Health Network, Toronto, ON Canada; University of Alberta, Edmonton, AB Canada; University of Cincinnati, Cincinnati, OH USA; Accreditation Canada, Ottawa, ON Canada; Alberta Health Services/University of Calgary, Calgary, AB Canada

**Keywords:** Medication management, Mixed methods, Human factors, Home care safety, Patient safety, Seniors, Caregivers, Family, Paid providers, Poly-pharmacy, Chronic illness, Complex care

## Abstract

**Background:**

Patient safety is a national and international priority with medication safety earmarked as both a prevalent and high-risk area of concern. To date, medication safety research has focused overwhelmingly on institutional based care provided by paid healthcare professionals, which often has little applicability to the home care setting. This critical gap in our current understanding of medication safety in the home care sector is particularly evident with the elderly who often manage more than one chronic illness and a complex palette of medications, along with other care needs. This study addresses the medication management issues faced by seniors with chronic illnesses, their family, caregivers, and paid providers within Canadian publicly funded home care programs in Alberta (AB), Ontario (ON), Quebec (QC) and Nova Scotia (NS).

**Methods:**

Informed by a socio-ecological perspective, this study utilized Interpretive Description (ID) methodology and participatory photographic methods to capture and analyze a range of visual and textual data. Three successive phases of data collection and analysis were conducted in a concurrent, iterative fashion in eight urban and/or rural households in each province. A total of 94 participants (i.e., seniors receiving home care services, their family/caregivers, and paid providers) were interviewed individually. In addition, 69 providers took part in focus groups. Analysis was iterative and concurrent with data collection in that each interview was compared with subsequent interviews for converging as well as diverging patterns.

**Results:**

Six patterns were identified that provide a rich portrayal of the complexity of medication management safety in home care: vulnerabilities that impact the safe management and storage of medication, sustaining adequate supports, degrees of shared accountability for care, systems of variable effectiveness, poly-literacy required to navigate the system, and systemic challenges to maintaining medication safety in the home.

**Conclusions:**

There is a need for policy makers, health system leaders, care providers, researchers, and educators to work with home care clients and caregivers on three key messages for improvement: adapt care delivery models to the home care landscape; develop a palette of user-centered tools to support medication safety in the home; and strengthen health systems integration.

**Electronic supplementary material:**

The online version of this article (doi:10.1186/s12913-015-1193-5) contains supplementary material, which is available to authorized users.

## Background

Patient safety is a national and international priority with medication safety earmarked as both a prevalent and high-risk area of concern [1, 2]. Medication safety requires the integrity of a complex series of interrelated steps, such that failure to adequately assess, prescribe, dispense, store, administer, and monitor medications can potentially lead to adverse events and harm. Human beings are fallible as they receive, transmit, and act upon information related to medication in the home care environment, which is not typically designed for providing health care and where most care is provided by unregulated workers, family, and unpaid caregivers [3, 4]. Yet, medication safety research has focused overwhelmingly on institutional based care provided by paid healthcare professionals, which often has little applicability to the home care setting. Some examples of medication-related problems in the home setting that are often peripheral to hospital settings include economic issues such as whether the individual can afford to fill his or her prescriptions; access issues, such as whether the individual has the physical capacity to get to a pharmacy; and social issues, such as living with an overwhelmed caregiver who has his or her own health concerns.

Caring for an individual with a chronic illness in their home is inherently complex. A rise in the medicalization of private homes, resulting not only from the escalating threshold for hospitalization but the increasing acuity of patients at the time of discharge, has been facilitated by the explosion of “hospital at home” services and the increasing availability of mobile technology (i.e., peritoneal and home haemodialysis, long term intravenous catheters and oxygen/inhalation therapy) [5]. Furthermore, the physical environment, family dynamics, the cognitive and physical abilities of the client and caregivers, are examples of other essential factors to be considered when delivering services. Caregivers are often elderly and must contend with their own health challenges. Stress and fatigue, with a lack of adequate preparation and education related to managing an array of medications, can also degrade the quality of care being provided over time. Caregivers often lack sleep as they provide around-the-clock care. This is in stark contrast to the institutional scenario where there are two or three shifts of professionals who provide around-the-clock care.

A recent systematic review of the home care literature related to medication management identified that individual knowledge, cognitive functioning, and poly-pharmacy were significant issues related to medication errors, and/or potential inappropriate administration of medication. Absent in the literature was evidence related to outcomes such as a sense of wellbeing, confidence to continue medication administration, and the experience of medication mismanagement from the perspective of the individual [6]. Also noted was a paucity of research into the perspectives and impact of family/caregivers regarding medication management even though we know from past research that client and caregiver safety are closely linked [7–11].

Given the lack of rigorous research to consistently identify issues related to medication management in home care, additional and replicable studies are needed to provide direction for interventions aimed at potentially mitigating the risks for both recipients and providers of home care [6].

Safety compromise in medication management can be extremely costly to individuals, their caregivers/families, providers, and the healthcare system [12–17]. A report by the Institute of Medicine on medication safety concluded that approximately 1.5 million preventable adverse drug events occur per year, resulting in a total cost of $3.5 billion US [18]. Canadian studies indicate that as many as one in five Canadians suffer adverse events following their discharge home from hospital, and two thirds of those events are related to compromised medication safety [17, 19, 20]. Furthermore, individuals themselves have identified problematic outcomes related to insufficient medication safety processes. The Commonwealth Fund International Health Policy Survey of Sicker Adults in Eleven Countries found that 5 % of 3958 people in Canada reported they had received the wrong medication or dose [21]. This critical gap in our current understanding of medication safety in the home care sector is particularly evident with the elderly who often manage more than one chronic illness and a complex palette of medications, along with other care needs. In addition, the challenges of documentation and communication, which are heightened at points of transfer across sectors [19, 21–23]; also increase the potential for inadequate medication reconciliation and its attendant risks. Providers can engage clients and family/caregivers in conversations and collaborate with them to mitigate risks; but even when this is done, the nature of the home setting requires clients and caregivers to regularly exercise autonomous decisions about medication use in the context of minimal professional supervision as well as frequently strained or absent home and community supports [24]. Thus, the care and safety of clients around medication management cannot be attended to without including the family members, unpaid caregivers, and paid providers in the equation [8–11, 24, 25].

## Methods

This study addresses the medication management issues faced by seniors with chronic illnesses, their family, caregivers, and paid providers within publicly funded home care programs. This paper focuses on the findings related to the following research questions:

1. What medication management issues do seniors with chronic illness, their family members, caregivers, and paid providers identify within publicly funded home care programs in Alberta (AB), Ontario (ON), Quebec (QC) and Nova Scotia (NS), Canada?
2. What socio-ecological factors contribute to or reduce the risks around medication management?
3. What strategies do seniors with chronic illness, their family members, caregivers, and paid providers employ to mitigate the risks of medication management at home in the these four provinces?

### Study design

The study methodology is Interpretive Description and multiple methods were used in data collection including participatory photographic methods adapted from previous research [26] to capture and analyze a range of visual and textual data. Interpretive Description (ID) was developed from Thorne’s experience using classic qualitative methods such as Grounded Theory and Phenomenology and quantitative methods in which her intent was to generate new insights for clinical practice as opposed to theorizing or measuring phenomena. ID is conceptualized as an approach comprised of three elements; the objective, the mechanisms, and the product [27]. In this study the objective was to generate new insights into medication management in the home care setting and the mechanisms included multiple methods of data collection. The product produced through data analysis was a set of patterns which considered together, offer explanations rooted in the data about the complexity of medication management for home care clients and their families. The patterns provide insights for clinical practice and directly inform the recommendations.

#### Theoretical perspective

Social ecology is the theoretical perspective underpinning the study design and methods. A Socio-ecological perspective means that humans and the environments in which they live are in constant interaction with one influencing the other socially, emotionally, functionally and physically. Socio-ecological thinking enables researchers to apply an ecological perspective to a phenomenon such as medication management and to describe humans in environments at several levels of aggregation: individual, family, organization, community, and population. A socio-ecologic perspective provides a framework for understanding the diverse personal and environmental factors and the interrelationships among these factors that influence a given health situation [28–30]. The identification of the multiple levels of the determinants of a problem facilitates the identification of recommendations for action across these levels.

Three successive phases of data collection and analysis were conducted in a concurrent, iterative fashion [31], which is described in detail in a previous publication [32]. In brief, our iterative approach entailed coding visual and textual data as we collected it to progressively inform our data collection and analysis within and between each successive phase of the research. Field notes recorded during each phase of the research also informed our data analysis.

This study took place concurrently in four Canadian provinces (AB, ON, QC, and NS) from 2009–2012. In our work, home care patients are referred to as “clients,” and the term “caregivers” signifies unpaid family, friends, or neighbours who support and provide care for the client. “Providers” are regulated or unregulated individuals who are paid to provide home care services to clients. Data were collected in eight urban and/or rural households in each province (*N* = 32). In total, 94 interviews were conducted with chronically ill seniors (*N* = 32) receiving home care services in each of these households, their family/caregivers (*N* = 33), and their paid providers (*N* = 29). Participating clients and caregivers self-identified themselves as Canadians from a variety of cultural groups (e.g., Italian, Polish, Jewish, Egyptian, British) and varied in socio-economic status. Educational level attainment ranged from Grade 8 to a University degree. Chronic illnesses in client participants included congestive heart failure, chronic obstructive pulmonary disease, cancer, diabetes, renal failure, and rheumatoid arthritis. Most clients were women with a mean age of 79 years (range = 67–92 years) while nearly half the caregivers were men with a mean age of 66 years (range = 32–93 years). In addition to the household interviews, focus groups were conducted with home care providers (*N* = 69) in each province. Providers were split evenly between regulated and unregulated home care providers (mostly health/personal support workers, Licensed Practical Nurses and Registered Nurses) who were predominantly women.

Ethical approval was obtained from the following Research and Ethics Boards: Dalhousie University, McGill University, L’agence de la santé et des services sociaux de Montréal, Centre de santé et des services sociaux Cavendish, University of Ottawa, Victorian Order of Nurses (VON Canada), Hamilton Niagara Haldimand Brant Community Care Access Centre, University of Alberta, Alberta Health Services, University of British Columbia-Okanagan. This study adheres to the qualitative research review guidelines (RATS) [33].

### Recruitment

This multisite study had team members that facilitated the recruitment of potential participants living in their respective province. Health agency personnel contacted prospective participants to determine their interest in the study. The researcher who explained the study, answered questions, and arranged an interview for those willing to participate. Informed consent was obtained prior to the interviews and focus groups.

### Data collection

In Phase 1, audio-recorded semi-structured interviews were conducted with clients and caregivers in their home. Although client and caregiver interviews were conducted separately (when possible) to allow for more freedom in speaking about their respective concerns, when participants preferred to be interviewed together their request was honored. In addition to the interviews, photo walkabouts [32] were conducted during which participants visually guided the interviewer through their home while describing their daily experience and routine including medication management. A digital camera and recorder were used to capture the concerns or strategies that were pointed out to the interviewer. Visual methods are increasingly being used in social science and qualitative research as a way to encourage participant collaboration while accessing the experiences and voices of research participants [34, 35]. Interviews with paid healthcare providers were conducted at a location of their choice that afforded privacy. Triads in qualitative research can be considered as “three participants interviewed as a set.” Linking the interviews of clients, caregivers, and providers has been shown to generate a richer understanding of study participant’s needs and experiences [10, 36].

In Phase 2, we used our analysis of Phase 1 data and selected images to conduct 1—3 photo elicitation kitchen table talks (KTTs) per province (*N* = 8) with clients, caregivers, and paid providers approximately one year following the initial interviews (See Additional file 1—Semi-Structured Interview Guide). Select images and text from the Phase 1 walkabout and interview data were used to stimulate dialogue. The KTTs enabled the researchers to observe participants and their inter-relational dynamics [37] in the setting where services were delivered, as well as to elicit and stimulate group discussion on any additional concerns or changes regarding medication management. The KTTs also provided an opportunity to test out patterns identified in the concurrent analysis of the visual and textual data.

In Phase 3, we used our evolving interpretation of the patterns to conduct two focus groups (one with regulated care providers and one with unregulated care providers) at the participating agencies in each of the four provinces. The focus groups served a dual purpose; first as a validity check to obtain feedback on the research team’s interpretation of the visual and textual data; second to hear from this experienced group of home care providers, most of who were not involved in the Phase 1 interviews, about safe medication management. An additional table file shows in more detail sample interview and focus group questions [see Additional file 2].

### Data analysis

Analysis was iterative and concurrent with data collection in that each interview was compared with subsequent interviews for converging as well as diverging patterns. The interviews, kitchen table talks, and focus group recordings were transcribed verbatim and managed with NVivo 9 software [38]. Initially, two researchers independently coded the textual data from each province, identified patterns, and the relationships among the patterns. Two members of the team, including a human factors engineer (HFE), coded the photo narrated data in a similar fashion. Open coding was used followed by focused coding with the development of the patterns. Open coding consisted of line by line coding. During focused coding, like codes were then clustered into groupings which were eventually articulated as distinct but inter-related patterns. In some instances the naming of the patterns was obvious as like codes were grouped; in others, further data analysis was required to name the pattern. Human factors analytic methods were employed to augment our understanding of how clients, caregivers, and home care providers experience the environments, equipment, and other tools (e.g. documentation systems) that were available to them as they manage their medications at home [39, 40]. In the case of home care, HFEs understand the need to examine all factors at play and the potential threats to quality and safety through the analysis of mismatches in provider/caregiver/client capabilities, care processes, medical devices and other equipment, and the physical environment in which care takes place [40]. In this study, three co-Principal Investigators analyzed data and met regularly to discuss the findings from the four provinces. The analysis team began by analyzing respective data sets by province to identify recurring, converging, and diverging patterns in the data. Despite some variation in the naming of patterns sets by province, in the final analysis respective coded data sets were mapped to six agreed upon patterns. The core research team, including knowledge users from each province, met with the analysis team to discuss and further validate the findings of the study.

## Results

Six patterns were identified that illuminate potential and real safety concerns in medication management in home care. Although there were some differences across provinces, the focus of this paper is on the remarkable similarities of safety concerns identified. These patterns intersect with each other, provide a rich portrayal of the complexity of medication management in home care, and were represented in all four provinces.

### Living with a variety of vulnerabilities that impact the safe management and storage of medication (Fig. 1)

Managing medications safely was inextricably linked between the client and their caregiver as each was dealing with multiple health issues and complex care regimes. Clients and caregivers alike revealed multiple sources of vulnerabilities such as cognitive impairment, dexterity issues, caregiver and other family member illnesses, and risky practices in the storage of medications that unintentionally or unknowingly impacted their ability to safely manage medication regimens.

Caregivers’ own health issues and the added stress caused by looking after another left both clients and caregivers in a vulnerable state and feeling isolated. Caregivers felt obligated to provide incrementally demanding care because there was often no one else available to do it. Homes with two older persons, both of whom often had chronic diseases and fluctuating levels of health, sometimes experienced role reversal with the ‘client’ taking on a caregiver role. Roles were determined simply by who was the sickest at the time.

Clients and caregiver sometimes forgot to take their medications, “Either he forgets…Sometimes I forget too…Our memories are not as good as [they] used to be” (client). In this household, the client’s medications were dispensed in a bubble pack system while the caregiver’s were dispensed in bottles. The caregiver would press the pills from the bubble pack onto a china plate for the client and she admitted that sometimes they fell to the floor. They kept the clients bubble packs in various plastic bags on the dining room table.

In another instance, a caregiver who did not live with the client shared that she often either found bottles of mixed medications that the client said he forgot or omitted to take or found medications on the floor. The client shared that he often omitted his diuretic medications if he went out for the day. In addition, reduced dexterity due to having a paralyzed left arm made it difficult for him to handle the bubble pack, medication bottles, and to pick up pills that fell to the floor, so he didn’t take those medications. Although he resided in seniors accommodation and received home care visits twice daily, his medication management and adherence were poor.

Storage of, and access to, medications in homes was variable. In one household, morphine elixir was stored in an unlocked kitchen cupboard. Young grandchildren often visited and were observed climbing up to the cupboard to retrieve the morphine for the caregiver to administer to the client during one of the researcher visits. When researchers asked if they ever considered child safety locks the caregiver explained, “They just climb on the counter to get it to be helpful… So you girls know not to touch that ever.” These examples illustrate that the vulnerabilities related to medication management present safety risks not only for clients and caregivers but also to their visitors.

Home care environments did not generally have built-in “ideal” storage locations for medical supplies and equipment. Multiple storage locations made adherence more challenging, medications often get lost in clutter and in cupboards. Figures 2 & 3 illustrate the storage of medications in two different homes.

**Fig. 1:**
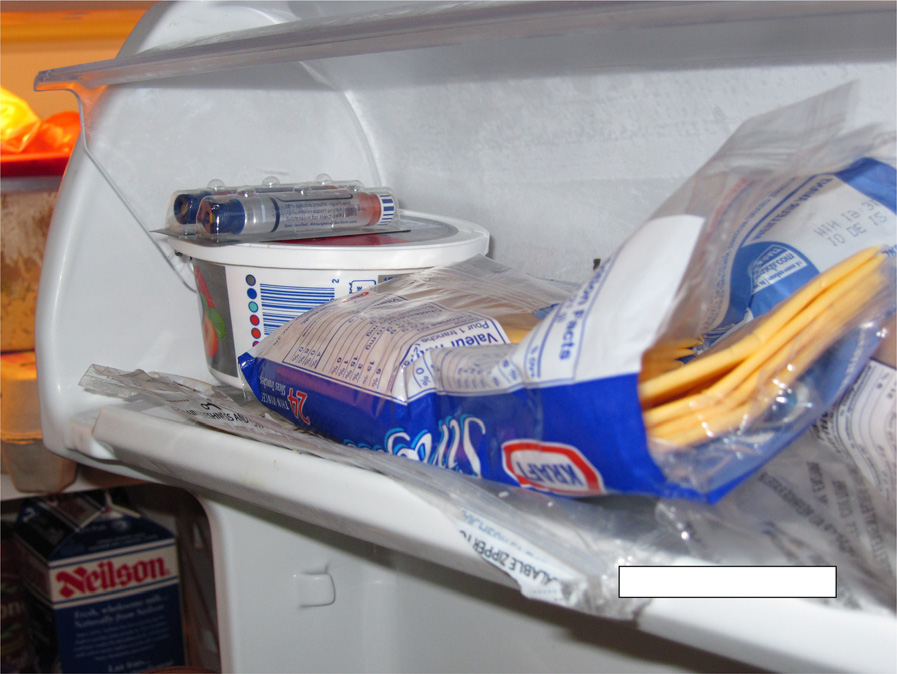
Vulnerabilities of medication storage

**Fig. 2:**
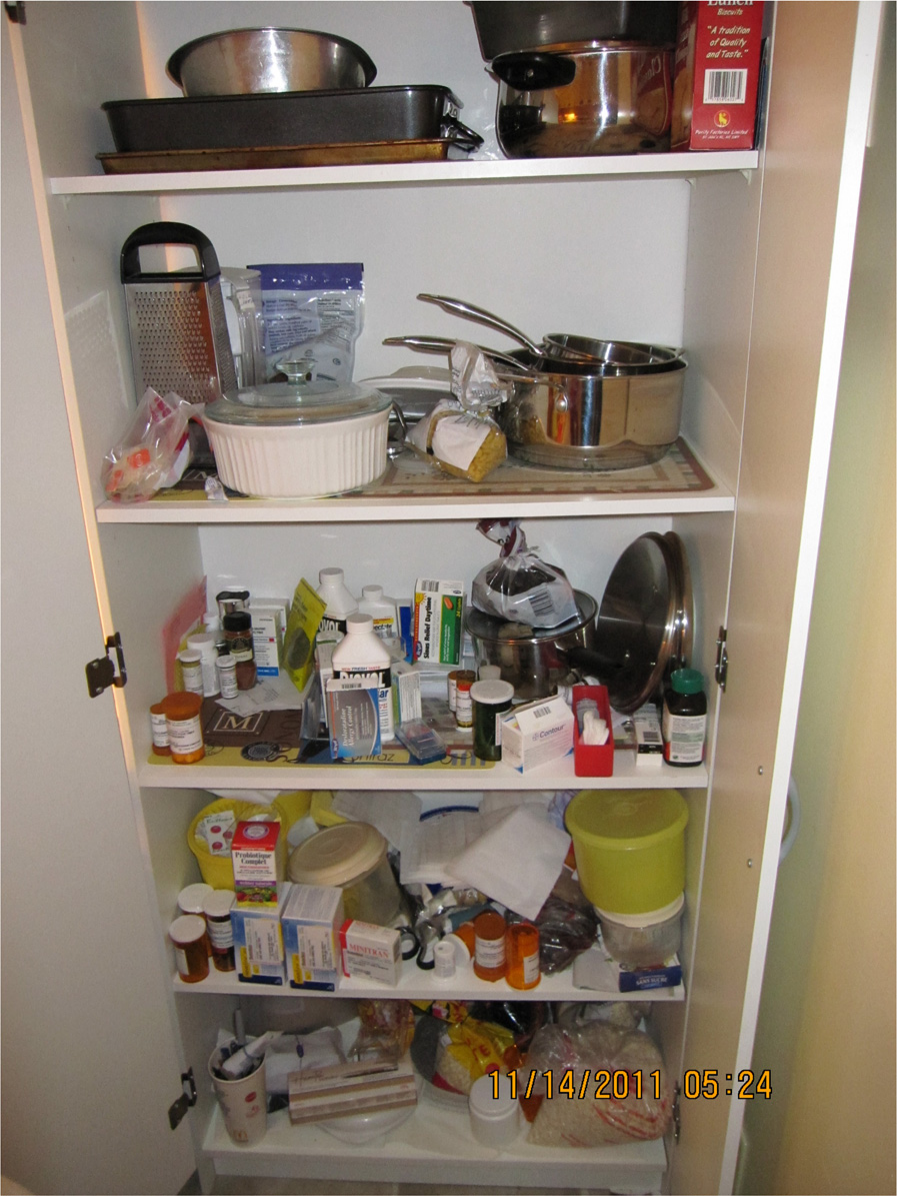
Medications stored with non-med related items in a cupboard

**Fig. 3:**
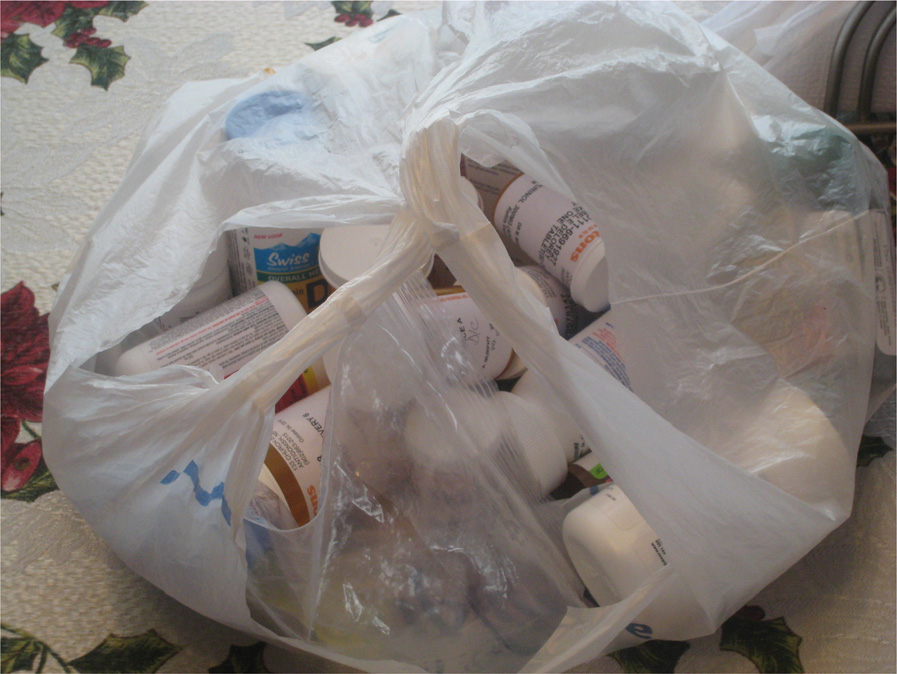
Medications stored in a plastic bag on a table

**Fig. 4:**
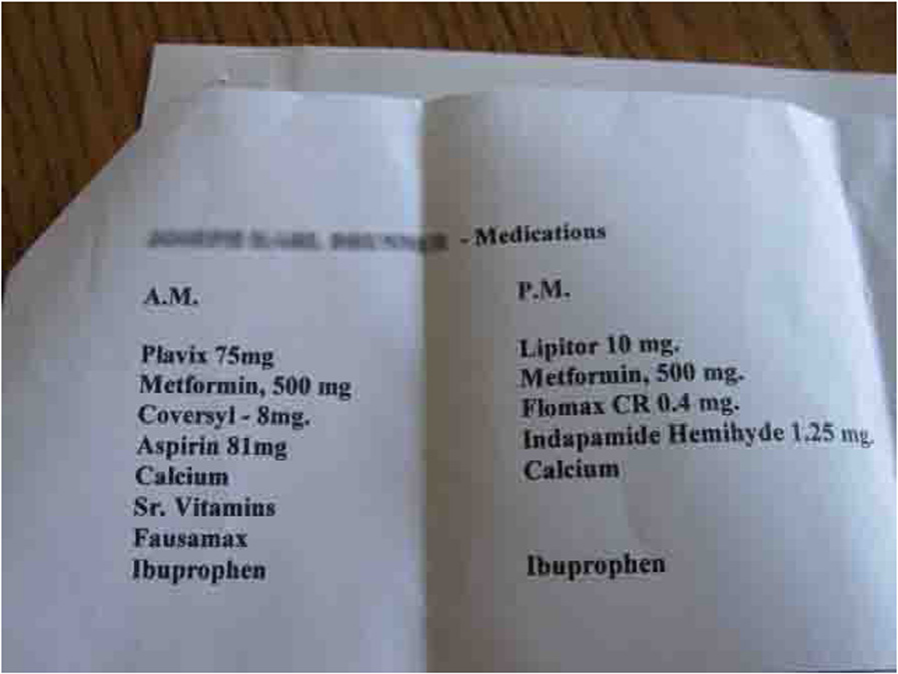
Caregiver tracking of husband’s medications on a computerized list

**Fig. 5:**
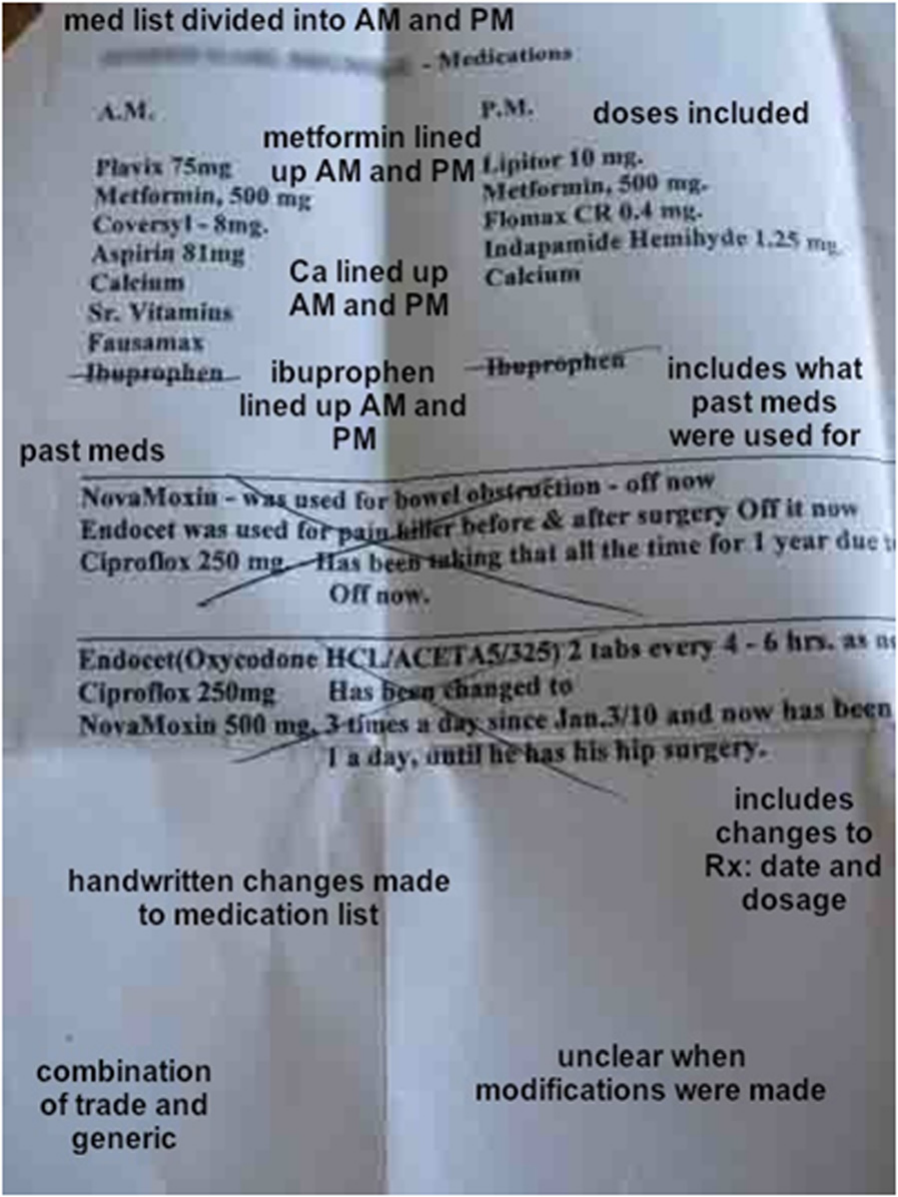
Caregiver’s record of clients medical and medication history

**Fig. 6:**
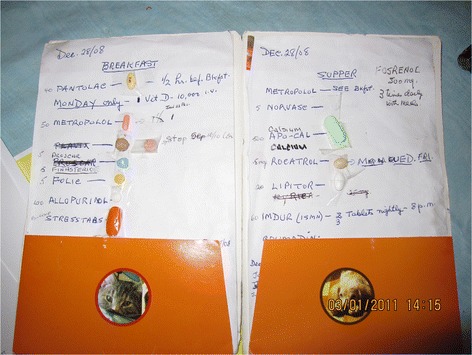
Client’s personal record of medications

**Fig. 7:**
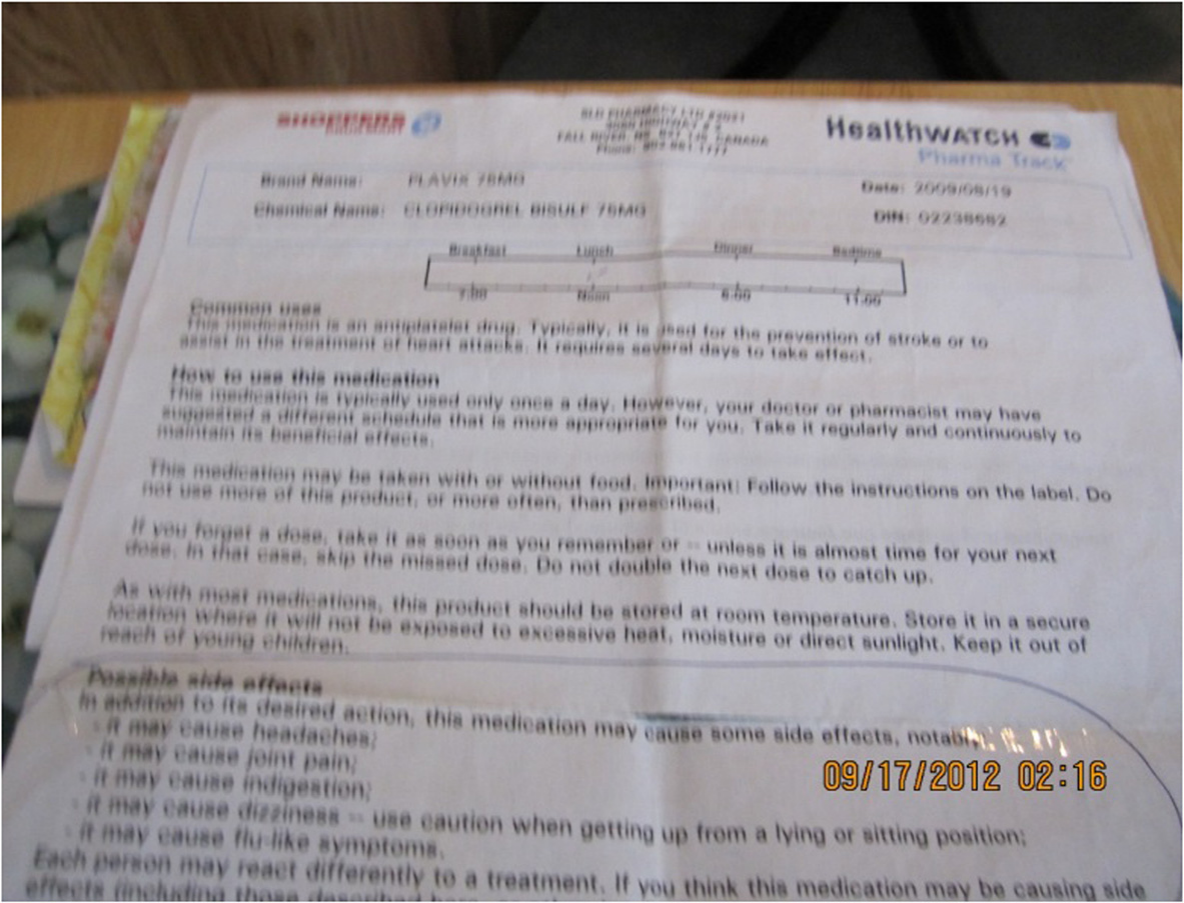
Pharmacy medication education material

**Fig. 8:**
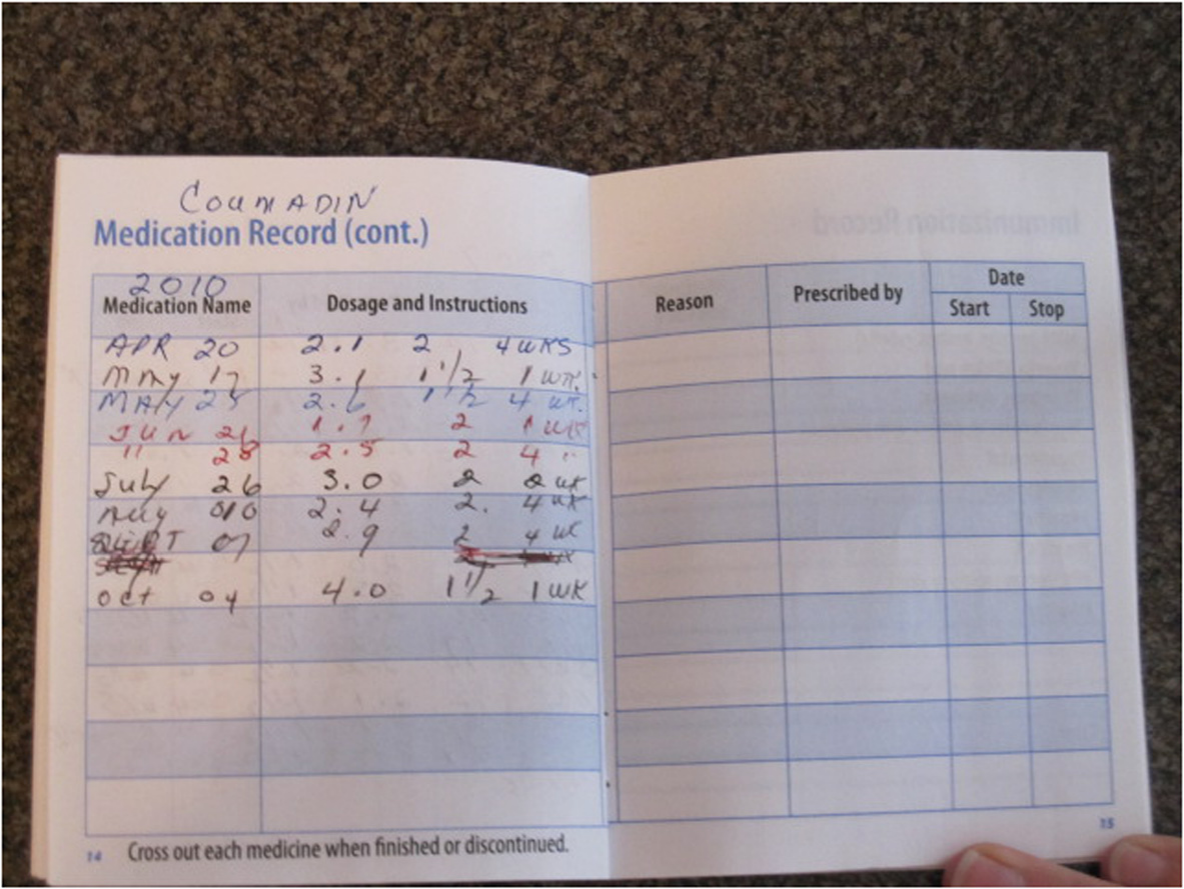
Client workaround to track Mean Normal Prothrombin Time (INR) and Coumadin doses without a home chart

Home care providers anticipated that clients and their caregivers would share the responsibility for their care, which included administering their medications, maintaining an adequate supply, safe storage, and at home treatments such as peritoneal dialysis. Caregivers often felt conscripted into providing care to the client. One said, “They [nurses] come, but they push… Why aren’t you doing it [client’s peritoneal dialysis]? And sometimes when they wouldn’t come, you know at until midnight four or five nights in a row, they would say well, if you were doing it [client’s peritoneal dialysis], then it wouldn’t matter, you could do it when you want it. So sometimes I felt that they were being late deliberately, because it is more of a push.” Although reluctant, this caregiver resisted pressure to manage her husband’s peritoneal dialysis until finally succumbing to the pressure to do so.

Providers’ assumptions about a caregiver’s ability to safely manage were not always accurate and occurred whether or not the caregiver and client lived in the same house, and whether or not the caregiver was able or willing to take on the care-giving responsibility. In one household, a provider explaining that the client’s son, a nurse, “doesn’t practice anymore but having the training at least definitely facilitates management… that was the reason I was okay with him staying at home because his son was there”. However, the provider was not aware that the son was no longer practicing nursing because his licence had been suspended due to substance abuse, placing everyone in a risky situation.

Another erroneous assumption by some providers was that the wealth of the household would enhance caregivers or clients’ ability to manage medication safely. As one provider expressed: “They’re quite wealthy. They live in a mansion so they have access to a lot. …I think if they needed any extra care put in place, they would definitely be able to sort that out.” However, the caregiver wife in question shared her concerns with researchers about how she was managing in general due to her increasing forgetfulness and tiredness, as well as her reluctance to ask family or home care providers for additional help. Healthcare providers were also unaware that the wife was taking her own medication for early onset dementia. These assumptions were not only inaccurate, but also left clients and caregivers in risky and unsafe circumstances.

Examples in this pattern of vulnerabilities revealed how cognitive impairment, dexterity issues, and caregiver illnesses impacted clients and caregivers abilities to safely manage medication regimens in their home. Assumptions about caregiver’s abilities to manage a client’s medications often placed clients and caregivers in risky situations. An additional table file gives further examples of vulnerabilities in more detail [see Additional file 3].

### Working to sustain adequate supports for medication related issues and other care requirements

This pattern provides evidence of the effort required by clients, caregivers, and providers to ensure systems that work. For example, in one household, the client could not remember when he took what. The neighbour “left them [medications] all out and he [client] took them. He had one of those Advair® puffers. It had like 88 doses and he [client] took them all in 24 h. It’s been a constant battle in that house.” In response to this challenge, a lockbox was used for storing medications. The neighbour, who was the caregiver, would get the medications out of the lockbox and set them out as prescribed so the client could self-administer. Several clients in the study as a strategy to support safe medication management and administration used lockboxes.

Clients and caregivers often relied upon family members if they had unanswered questions about medication times or side effects, where to go for further advice, or how to obtain home care services. One caregiver reported, “We asked his son to help us because he’s a nurse” and “Our granddaughter is also a nurse, and right away, she’s the one that goes online and she sends me [caregiver] the information. So actually it’s not me going online, it’s actually my granddaughter”.

In some cases, the caregiver refused offers for additional home care support. Providers often respected the client and caregiver decision to decline more support and knew that ongoing deterioration in the client or caregiver’s health would eventually result in further supports being needed. As one provider explained, “I have suggested that they get the medications done up in a Dosette® so then she doesn’t have to worry about it. But so far they’re not interested.” In contrast, some caregivers reported feeling that they simply were not heard when they requested additional support. During a focus group with registered professionals, one participant said that they knew that at times their colleagues did not listen to the caregivers, stating: “They got home care and they were trying to tell the nurse that they weren’t coping, and that she [client] wasn’t getting better, she [client] was just getting worse and the provider just decided everything was fine”. The continuous effort by clients and caregivers to manage medications was arduous in some households.

Clients and caregivers also received support in specialized clinics. This included access to multiple health professionals who were able to review the client’s condition and medications on a regular basis, address medication related concerns promptly, and in some cases coordinate services. Getting support for medication management and other chronic health concerns in one place increased client and caregiver satisfaction and sense of security. One couple reported, “If we need renewals, we don’t go to the doctor, we do it at dialysis. They do it for you…They are on top of everything you know… Bad indigestion, constipation, or whatever, they will do something about it. If they themselves can’t handle it or don’t have results, they will put you on to the specialists in the hospital and it becomes easier for us to get into them”. Conversely, personal support workers and nurses shared during focus groups that multiple reviews and prescribing of medications created difficulties for home care staff in terms of keeping track of changes.

Changes to medications by a clinic physician were seldom relayed to the home care case manager, which caused confusion. It was evident that communication about medications during transitions of care often broke down.

### Varying degrees of shared accountability for care

Shared accountability refers to the capacity for clients, caregivers and providers across the health system to collaboratively plan, implement, and periodically re-assess the safety of medication regimens and related care to ensure optimal outcomes. However, there was wide variation between households regarding how successfully team members, including clients and families, were able to collaborate for safe medication management. Figure 4 is an example of a caregiver tracking her husbands medications on a computerized list.

At times, participants attributed challenges in sharing accountability to factors beyond their individual control. Providers expressed that discontinuing home charts (that used to be shared among all providers and agencies) with palliative clients decreased opportunities for collaborative engagement in care planning. Providers were frustrated with a poorly functioning medication reconciliation system that was often ignored or not completed by the family doctor. Open dialogue between various team members was at times sufficient to establish a workable sharing of accountability. Other times, it was very unclear, or contested ground. One provider observed that medication re-orders could be missed when a client forgot to call them into the doctor, but that this particular client preferred to retain that function because “she does like to have some contact with the doctor and do that herself.” In another household the accountability for care was eventually agreed upon from the provider perspective, but only after considerable dialogue with reluctant family members: “So four times a day we got the family calling…to tell her [client] to do her insulin and blood sugar, to take certain tablets… And they weren’t buying that at first, they said, you know we haven't got time for this. And I go, this is your mother, you know, do you want her to stay at home or do you want her to go to a nursing home? So they sat down and had a conference with the family and they agreed. They put speed dial on all their different phones and different people were responsible, and that’s all it took for this woman, is to get a phone call from a child”.

Clients, caregivers, and home care providers described the importance of being consciously committed to taking the time and effort to optimize medication safety in a variety of ways. One client indicated, “You know, I check the INR every Monday.” One provider described the considerable effort she expended to ensure that her client’s pain management regime got back on track:

“I had a man that stopped all his [narcotic] medication and we had to start his medication again … he had such bad withdrawal that we didn’t want to wait until the evening for his next long acting narcotic …So he took his long acting right away because I spoke to the pharmacy and to the doctor about how to start everything again. Then we wrote down together me, and his wife, and the patient what times he was going to take his medications to bump it back to a regular twelve hourly medication…then make that very clear in the notes so that they can reinforce that and make sure that that’s happening…. And then I left a voice message with that nurse about what was going on.”

Overall, engaging in shared accountability for medication safety was multi-faceted and unique for every household and their respective health care teams. Regardless of the source(s) of shared accountability, common elements of successful engagement observed in this study included consistent, reliable relationships, the willingness to problem solve with others to find safe solutions, and the ability to see what needed doing and follow through. Identified barriers to effective shared engagement included constantly changing home care providers, the use of multiple physicians and pharmacies who did not communicate with each other, and a lack of client-friendly mechanisms such as home charts, education focussed visits, and shared care plans for households and providers to design safe medication strategies together.

### Households put systems of variable effectiveness in place to manage medications

Home care clients and families put a variety of systems in place to manage medications with varying levels of effectiveness. They described this as “having a system.” Some participants used highly organized systems, others less so, and some were struggling to figure out what medication to take when. Most participants reported they were given a list of their medications upon departure from hospital with the dosage and times, and from this, they figured out how they would proceed.

Each home differed in their management of getting prescriptions filled, renewed, and delivered, packaging of medications, taking of medications, and storage. Some participants used computers and other devices to organize their medications. One caregiver described creating a medication document that was easy to edit and update, as well as accessible to print out for appointments and admissions to health service facilities: “I decided to start keeping a record of his medical history on the computer, so now when something happens I just add to it and leave it on that. And then with his medication, they always ask that too [emergency and hospital staff on his frequent admissions] so I had the pills here, to put down the right amount of milligrams”. Figure 5 is a record of a clients medical and medication history.

Despite the limitations caused by their illnesses, clients would often create effective systems. One client who was completely home bound developed strong relationships with the physician and pharmacist. She managed to get multiple prescriptions, including narcotics renewed, packaged, and delivered by a good friend and caregiver. This client kept a diary of all healthcare related activities including changes to medications and instructed her caregiver regarding her schedule. With the exception of her refrigerated insulin, she kept all her medications in one designated area (command post) in the kitchen by the window for good light. She adjusted her anticoagulants based on regular laboratory reports obtained from her doctor, and applied and removed nitro patches daily along with taking several other medications. The result of being cognitively alert, and having problem solving skills as well as such an efficient and organized system, was that this client, despite being very physically debilitated, managed to live out her life in her own home. Figure 6 is an example of a clients personal record of medications.

In contrast, some clients felt it was not their responsibility to manage their medications or “have a system.” “Sometimes the people [health providers] will say, you know, you can do all that, you can set up the machine, and you can do the exchange. And I still say if you’re training me to be a nurse, you’ll have to pay me. You know, I’m no nurse”.

### Navigating for an optimal medication regimen requires poly-literacy (health, medication, and healthcare system literacy)

The pattern of poly-literacy encompasses the varied knowledge that participants held about their medications, their health conditions, and the resources they could access to support their medication management efforts. Some clients did not understand the purpose of their medications, how they acted on their illness, and often did not recall receiving explanations related to either: “I get confused. There are so darn many medications I’m just a walking drugstore…I do not know enough. I would like to know more but it seems there is never enough time for this.” A caregiver reported, “They [providers] don’t go over what medications do inside the body.” Similarly, a provider commented “I think often they [clients] go to a physician and they don’t understand, they [clients] say they [physicians] don’t have time for them…The clients say: “He [physician] just handed me this pill. I don’t know what it’s for.” He said, “Take this pill.” Some clients had their own interpretations of medications and directions around taking them. These interpretations did not always match what the original directions on the prescription, as one caregiver notes: …”she [client] has a certain interpretation of how she should take her medications, and she has a lot of them. And she takes them somewhat haphazardly. So she has difficulty following through with the protocols for her puffers, anything that has to do with her asthma and antibiotics.” Written materials, although received by clients were not always useful. One client explained, “I usually do receive sheets from the pharmacy but I get kind of disgusted because from a layman’s point of view, when you read this you’d be scared to take a drink of water. You know, it’s so many things that they list there that you don’t know whether to be scared or not. Figure 7 is an example of Pharmacy medication material.

Home care recipients varied in their interest and engagement related to their illness and medications, while healthcare providers reported significantly limited time to provide client/family education. The data highlighted the need to further understand the extent to which healthcare professionals believe they could engage care recipients in learning more about their medications as well as their health conditions and the resources they could access to support their medication management efforts.

### Systemic challenges to maintaining medication safety in the home

Several systemic challenges to safe medication management beyond the control of individual home care clients, caregivers, or providers were identified. In many instances, clients, caregivers, and providers resorted to workarounds to try to improve medication management despite system barriers. For example, a common strategy to deal with frequent medication changes in households using blister packs was to manually remove the discontinued medication and then re-tape the blister pack shut. A workaround devised by some home support workers to identify poorly labelled medications was to tape each pill to the back of the medication list in the home.

Issues pertaining to redundant documentation, missing documentation, and/or a lack of user-friendly documentation tools were a great source of frustration. Clients often devised their own tracking systems in the absence of better alternatives. Providers felt that the lack of a home-based client chart was detrimental to the continuity and quality of medication management. Figure 8 illustrates a clients workaround to track their Mean Normal Prothrombin Time.

One home support worker’s workaround to deal with this documentation gap was to make handwritten post-it notes when dispensed medications did not match the order and then call the home care nurse, to alert them to the addition of the handwritten post it note.

Providers doubted the reliability of using an unofficial communication log to keep each other updated on client changes in the absence of an official home based client chart. Some home support workers voiced their dissatisfaction with having to “chase” down missing discharge summaries to conduct medication reconciliation, indicating that in some instances, there were “months’ worth of sheets” that had not been picked up by the nurses who were legally responsible for overseeing the client’s medications.

Several potential or actual disruptions to safe medication management related to transitions in care. Providers asserted that elderly clients who were discharged from the hospital on weekends often went home with prescriptions for new medications, which they were unable to get out of the house to fill in a timely fashion. Some providers indicated that medical residents who prescribed new medications during emergency department visits were often hard to locate after clients returned home, and frequently could not recall the clients even if contacted.

Another systemic issue that most providers associated with medication safety was their own chronically heavy workloads. Providers described being rushed and distracted due to tight scheduling of visits restricting their ability to conduct timely re-assessments, joint care planning, and client and family teaching. As client acuity and overall caseloads escalated in home care, it became increasingly more difficult to adhere to best medication safety practices.

Providers, clients, and caregivers all described the challenges of having to deal with multiple providers, agencies, and organizations. Some examples included not having sufficient home care staff to ensure consistent client assignments or clients who had difficulty accessing a family physician resorted to an array of doctors in emergency departments, Medi-clinics, and a private for profit house call service to obtain new prescriptions or medication refills.

Taken as a whole, the systemic challenges to optimizing medication safety were numerous and wide-ranging resulting in workarounds being devised to try to mitigate the impact of a given system barrier.

## Discussion

The findings speak to the need for policy makers, health system leaders, care providers, researchers, and educators to work with clients and caregivers on three key messages for improvement around safe medication management in home care:

### Key message # 1 adapt care delivery models to the home care landscape

How care is assessed and delivered for clients and their caregivers within their respective household contexts needs to be addressed and questioned. In this study, each home harbored various vulnerabilities that affected the client and/or caregiver’s ability to safely manage their medications. With respect to medication literacy and the cognitive functioning of home care recipients, these findings mirror those of Godfrey & colleagues (2013) who found that individual knowledge and cognitive functioning were significant issues for medication management and medication errors. Effective approaches to safe medication management in home care therefore need to adapt to the complex reality that each home is a unique, unregulated private space where seniors and their caregivers manage complex medication regimens with limited training and education, which may increase the risk of a medication error [18]. This scenario constitutes a profound contrast to public institutions where regulation of the setting, practice and all care providers is multi-layered and overt.

This study provides additional empirical support for the notion that client safety is inextricably linked to the safety of the family/caregiver [8–11, 24, 25]. Caregivers, who were predominantly elderly in this study, found themselves taking on increased tasks and responsibilities while trying to balance their own commitments and pre-existing physical and mental health challenges. In addition to being on call 24-h a day, caregivers’ responsibilities often extended for weeks and even months without breaks or respite.

These findings confirm related work that queries the sustainability of home care delivery systems, which rely on the chronic overloading of numerous caregivers and providers [8–10, 41]. It follows that initial and ongoing home assessments need to evaluate not only clients’ and caregivers’ health conditions and physical capacities, but also pay close attention to the cognitive, emotional, social, functional, financial, and literacy capacities within the household, as well as their networks of support and willingness to accept responsibilities [10, 11]. Home care providers need to be responsive to the capacity, limits, and more importantly, the willingness of clients and their family caregivers to safely manage chronic conditions and medication issues in the home. Regular re-assessment and focusing on the client and caregiver(s) as the “unit of care” should be part of home care including updating and adapting care and supports to *their* changing health needs [4, 11].

The diversity of engagement between households in this study raises questions about how to best involve recipients and providers in collaborative models of safe medication management at home. While regulatory bodies for pharmacists, nurses, and physicians cite expectations around patient education, meaningful involvement of these providers in supportive medication safety assessment and education for clients and caregivers was highly divergent in this study.

Involvement ranged from ongoing, active efforts to no evident activities to enhance household literacy about medication safety. Given that individual knowledge, cognitive functioning, and poly-pharmacy are significant issues for home care safety [6], it seems timely to recommend consistent home care providers and one consistent community pharmacy for home care clients.

Medication and system literacy levels varied widely amongst participants and their ability to navigate the healthcare system. This variability needs to be accounted for when designing care models for medication safety. Since clients and family caregivers regularly make autonomous decisions about medication management at home with little or no professional support [7], functional levels of poly-literacy are essential. However, while access to medication safety information and advice should be readily accessible, many lack the education and resources to gather the information they need. Quality home care requires a model driven by communication and coordination with the flexibility to accommodate the needs of individual clients as well as family/caregivers [42]. Despite low literacy levels in most parts of the country [43], home care delivery models that enhance understanding for all recipients on how best to self-manage their care and to avoid hospitalization and institutionalization wherever possible need to be developed. The complexity and diversity of home care environments requires policy-makers, researchers, and other stakeholders such as community pharmacies, pharmaceutical companies, and human factors engineers to work more closely with clients, caregivers, and front line providers to create safer, more user-friendly medication management systems.

Adaptive models of home care delivery require timely case management. If home care models are not designed or funded to provide for timely case management, then meaningful gains to medication safety may remain elusive. Change should address consistent care providers, sufficient access to other vital supports such as respite care, primary care (including more use of inter-professional primary care teams), specialist nurse practitioner and physician care, and sufficient clinical pharmacist monitoring. These kinds of systemic changes require committed resources, targeted policies, and selective legislative support at the provincial and federal levels.

### Key message # 2 develop a palette of user-centered tools to support medication safety in the home

Across the four provinces, medication management systems varied in effectiveness. Diverse in-home workarounds were prevalent and not infrequently problematic. For instance, while certain household documentation workarounds appeared to work well, providers questioned the reliability of some strategies that caregivers and clients crafted to support safe medication management. Unique systems of varying safety were described for filling prescriptions, renewing and delivering them, packaging medications, and storage. Several providers suggested that shared tools with clients and caregivers such as a home chart would be more useful to encourage engagement, understanding, and shared accountability than many of the homemade documentation systems and other workarounds that their clients were currently trying to manage. Home care personnel, human factors engineers and other team members need to collaborate with clients and caregivers to develop and implement a palette of user-centered tools designed to meet the needs and limitations of seniors and to support medication management in the home [44]. Clearer drug packaging and labelling, user-centered education materials, shared, home-based charting systems, would strengthen clients’ and their families’ ability to safely manage their medications, while reducing preventable risks such as unsafe workarounds and haphazard medication tracking systems. Further research on the array of workarounds to cope with gaps in the health system is also warranted. The variety and creativity of systems that clients and caregivers devised to manage their medications ranged from remarkably efficient tracking systems to observed unsafe storage solutions. It may be impossible to have a universal standard of medication management in the home due to varying personal factors. Nonetheless, human factors experts are valuable resources who could study home-based medication management workarounds and collaborate with information technology experts, across the continuum of care (home, acute, primary, rehabilitation), community pharmacies, regulatory bodies, clients and caregivers to design shared written medication safety care plans, cross-sector medication reconciliation processes, and other user-centered documentation tools and delivery systems that improve medication safety.

### Key message # 3 strengthen health systems integration

Policy makers, practitioners, and funders need to work with researchers, recipients, and providers of care to study and narrow additional gaps amongst health sectors. These gaps include issues with transitions in care, lack of a functioning integrated electronic health record, communication failures between hospital, home, and community providers within and externally to the publicly funded system, and current care delivery models that hamper communication across sectors of care.

It is not realistic to expect medication safety to significantly improve for home care clients if challenges with system integration are not effectively addressed [45, 46]. For instance, findings from this study converge with other evidence where disruptions to transitions in care are frequently associated with communication gaps about medication-related issues [1, 17, 19–21, 47, 48]. Recent research by Romagnoli and colleagues in the U.S. supports this finding, noting multiple unmet needs related to medication safety for geriatric patients during the immediate post-hospital discharge period [48]. Integrated models of home care delivery, which focus on strengthening the continuity of care between sectors, merit investigation in future research.

Could the deployment of more home care nurse practitioners and clinical pharmacists specializing in the proactive community management of seniors with complex needs reduce physician visits and/or emergency and hospital admissions and client harms due to medication-related adverse events? Does the use of specialty clinic nurses and gerontological practitioners support clients and caregivers with progressive chronic disease to avoid medication-related incidents? Is it feasible to implement a community pharmacy notification system [18] that reduces the number of adverse events related to delays with starting newly ordered medications after discharge from hospital to home care? What can we learn from care network exemplars in Canada or in other countries, such as the UK’s *Improving the Future for Older People* [49], to reduce unnecessary emergency room admissions that are so often linked to issues with transitions in care? What are the effects of such systematic interventions on morbidity, mortality, and health system costs? Timely pursuit of these kinds of research questions would be very useful. The number of instances where inter-professional communication gaps were found to hinder medication safety suggests there is considerable room to enhance the delivery of effective team-based home care as well as the safety of handovers of emergency patients and in-patients to home care. However, while the critical role of handovers in safe care transitions is now recognized [19, 22, 23, 50], our findings indicate that home care clients, families/caregivers and providers need to have greater input into the formation of user-centered, safer handover and transition protocols. Furthermore, our findings support Moore’s point that even the most organized handovers require organizations and providers to value and allocate adequate time [51], a resource that Canadian home care providers are often taxed to find [52]. The roles of electronic health records, computerized decision support systems, and portable electronic devices also need to be studied further for their potential to strengthen the safety of handovers and other communication in home care [51, 53]. Pharmacies need to take a more active role to ensure timely, accurate communication amongst pharmacies, and home care providers. Participants advocated for the creation of accessible online updates of clients’ medications, while stressing the need to ensure that online notification systems replace rather than redundantly add to paper-based documentation systems. Medication changes do not always show up on electronic systems in a timely manner, and therefore could not be treated as a reliable source of continuously updated information. Hospital and community physicians, nurses, pharmacists, along with clients and caregivers need to be brought together with accrediting bodies, regulatory bodies, patient safety organizations, health sciences educators, researchers, and health ministries to explore mutually feasible ways to improve shared engagement in safe home medication management. A recent systematic review of handover education interventions in medicine and nursing [54] also indicates that reducing adverse events in health care, including medication-related harms, requires health professional programs to heighten their efforts to educate students for more effective communication and safer care. A number of households employed system workarounds to meet immediate medication needs in ways that could inadvertently sabotage medication safety. Some households tapped into other sectors of the healthcare system, such as specialty clinics, to renew their prescriptions when regular providers were not available, which often left various home care providers uninformed of medication changes. These scenarios illustrate how system integration initiatives such as medication reconciliation tools need to be coordinated with other system reforms for optimum benefits to be achieved.

### Limitations

The participants recruited for this study were required to have an unpaid caregiver. This criterion eliminated the participation of many elderly individuals who were living at home alone, and who manage multiple medications. Although the sample was somewhat diverse, it was limited to participants who could speak either English or French. Qualitative researchers must be mindful of the possibility of participants providing responses they believe the interviewer is seeking rather than reporting their actual experience.

## Conclusion

The findings from this study both complement existing research on medication safety in home care [4, 18] and provide new insights. This study illuminates the insights and experiences of clients, family/caregivers and providers who work together to manage multiple medications in the home. In conjunction with the evidence to date on the human and material costs associated with medication-related adverse events [12–17], our findings support the merits of quality improvement work to enhance medication safety through evidence-informed adaptation of care delivery models to the home care landscape. Future research should be aimed at developing a palette of user-centered tools that support medication safety in the home. Finally, strengthening health systems integration would enhance medication safety and medication-related client and caregiver outcomes in the home care setting.

## Additional files

Additional file 1:
**Semi Structured Interview Guide.** (PDF 191 kb) Additional file 2:
**Table of sample Interview & Focus Group Questions.** (PDF 171 kb) Additional file 3:
**Table of further examples of vulnerabilities.** (PDF 204 kb)

## Abbreviations

AB: Alberta
HF: Human factors
ID: Interpretive Description
INR: International Normalized Ratio/Mean Normal Prothrombin Time Prothrombin time
KTTs: Kitchen table talks
NS: Nova Scotia
ON: Ontario
QC: Quebec
RATS: Qualitative research review guidelines
