# Supplementary material for: Seniors managing multiple medications: using mixed methods to view the home care safety lens
Source: BMC Health Serv Res. 2015 Dec 12;15:548. doi: 10.1186/s12913-015-1193-5 (PMC4677040; doi:10.1186/s12913-015-1193-5)
Supplement: Additional file 1: — Semi Structured Interview Guide. (PDF 191 kb) [file 12913_2015_1193_MOESM1_ESM.pdf]

Seniors Managing Multiple Medications: Using Mixed Methods to View the Home Care Safety Lens

Phase 1

Semi-Structured Interview Guide for Clients

Title of Research Study: **Safety in home care: Focus on medication management**

Co-Principal Investigators: Dr. Ariella Lang, VON Canada, Dr. Patricia Marck, University of Alberta, Dr. Marilyn Macdonald, Dalhousie University.

1. Would you please tell me about the medications you are taking

Probes: What are they for?
Who helps prepare your medications (i.e., family member, providers, etc.)?
Is there anything special that needs to be done (i.e., crushing, taking with food, etc.)?
Are you taking anything else that is not prescribed (i.e., herbal supplements, vitamins, items from a health food store, etc.)?

2. Does anyone review your medications on a regular basis with you? Could you please explain, or provide an example?

3. Do you feel you know what you need to know about the medications you take? Could you please explain.

Probes: Does your home care provider talk to you about your medications?
Do you or your family member read the information pamphlets that come with your prescriptions or medications?

4. What happens if you forget to take a medication?

Probes: How do you tell if you have forgotten?
What happens if you take too much of a medication? How do you tell?

5. How do you get your prescriptions?

Probes: Who calls them in? Who brings them home?
How many doctors/nurse practitioners/specialists write prescriptions for you?
Do you get your prescriptions from the same pharmacy or pharmacist each time?
What do you do about refilling your prescriptions?
Do you cut down on your medications to make them last longer?
What do you do with outdated prescriptions?
Have you ever not filled a prescription for financial reasons (i.e., too expensive/could not afford)?

6**.** Has anyone in your house ever taken your medication, or have you ever taken someone else's medication, either by accident or not?

Probes: Do you ever share your medications with anyone?

7. Do you ever worry about having medications in your home? Please explain.

8. What kind of strategies or tricks do you use to remember to take your medication or to remember something important about a medication?

Probes: Do you use any kind of container to sort your pills in? Tell me about it and how it works.

9. Is there anything else you would like to discuss in relation to your medications? Have we left anything out?

Seniors Managing Multiple Medications: Using Mixed Methods to View the Home Care Safety Lens

Phase 1
Semi-Structured Interview Guide:
Family Members and Caregivers

<u>Title of Research Project:</u> Safety in home care: Focus on medication management

Co-Principal Investigators: Dr. Ariella Lang, VON Canada, Dr. Patricia Marck, University of Alberta, Dr. Marilyn Macdonald, Dalhousie University.

1. Would you please tell me about the medications the client is taking?

Probes: What are they for?
Who helps prepare the medications (i.e., the client, yourself, providers, etc.)?
Is there anything special that needs to be done (i.e., crushing, taking with food, etc.)?
Are you aware of anything else the client is taking that is not prescribed (i.e., herbal supplements, vitamins, items from a health food store, etc.)?

2. Does anyone review the client's medications on a regular basis with you? Could you please explain, or provide an example?

3. Do you feel you know what you need to know about the medications the client takes? Could you please explain?

Probes:Does the home care provider talk to you about the client's medications?
Do you or the client read the information pamphlets that come with your prescriptions or medications?

4. What happens if the client forgets to take a medication?

Probes:How do you tell if they have forgotten?
What happens if the client takes too much of a medication? How do you tell?

5. How do you get the client's prescriptions?

Probes: Who calls them in? Who brings them home?
How many doctors/nurse practitioners/specialists write prescriptions?
Do the prescriptions come from the same pharmacy or pharmacist each time?
What do you do about refilling the prescriptions?
Do you know if the client ever cuts down on medications to make them last longer?
What do you and the client do with outdated prescriptions?
Have you ever not filled a prescription for financial reasons (i.e., too expensive/could not afford)?

6. Has anyone in your house ever taken the client's medication, or someone else's medication, either by accident or not?

Probes:Do you or the client ever share your medications with anyone? With each other?

7. Do you ever worry about having medications in the client's home? Could you please explain or provide an example?

8. What kind of strategies or tricks do you use to help yourself and the client remember to take their medication or to remember something important about a medication?

Probes:Do you use any kind of container to sort the client's pills? Tell me about it and how it works.

9. Is there anything else you would like to discuss in relation to your family member's/the client's medications? Have we left anything out?
