# Supplementary material for: Seniors managing multiple medications: using mixed methods to view the home care safety lens
Source: BMC Health Serv Res. 2015 Dec 12;15:548. doi: 10.1186/s12913-015-1193-5 (PMC4677040; doi:10.1186/s12913-015-1193-5)
Supplement: Additional file 2: — Table of sample Interview & Focus Group Questions. (PDF 171 kb) [file 12913_2015_1193_MOESM2_ESM.pdf]

Additional File 4

Table 4 Samples of interview and focus group questions

**Client & Caregiver Interview questions**
**1.Would you please tell me about the medications you are taking**<br>• What are they for? Who helps prepare your medications (i.e., family member, providers, etc.)?<br>• Is there anything special that needs to be done (i.e., crushing, taking with food, etc.)?<br>• Are you taking anything else that is not prescribed (i.e., herbal supplements, vitamins, items from a health food store, etc.)?
**2. Does anyone review your medications on a regular basis with you?**<br>• Could you please explain, or provide an example?
**3. Do you feel you know what you need to know about the medications you take?**<br>• Could you please explain? Does your home care provider talk to you about your medications?<br>• Do you or your family member read the information pamphlets that come with your prescriptions or medications?
**4. What happens if you forget to take a medication?**<br>• How do you tell if you have forgotten?<br>• What happens if you take too much of a medication? How do you tell?
**5. How do you get your prescriptions?**<br>• Who calls them in?<br>• Who brings them home?<br>• How many doctors/nurse practitioners/specialists write prescriptions for you?<br>• Do you get your prescriptions from the same pharmacy or pharmacist each time?<br>• What do you do about refilling your prescriptions?<br>• Do you cut down on your medications to make them last longer?<br>• What do you do with outdated prescriptions?<br>• Have you ever not filled a prescription for financial reasons (i.e., too expensive/could not afford)?
6. **Has anyone in your house ever taken your medication, or have you ever taken someone else's medication, either by accident or not? Do you ever share your medications with anyone?**
**7. Do you ever worry about having medications in your home?** Please explain.
**8. What kind of strategies or tricks do you use to remember to take your medication or to remember something important about a medication?** Do you use any kind of container to sort your pills in? Tell me about it and how it works.
**Focus Group Questions**
**1. Would you please describe what you perceive to be the medication management issues that your clients face?**
**2. With your clients, are there any particular medications that concern you? Please explain, or provide an example.**
**3. Do you ever get the sense that your clients are not using their medications as instructed**
**4. Can you tell me what major medication changes you have witnessed in homecare in the past 5 years?**
**5. Do you think there are factors in your clients' homes that facilitate the management of their medications** (i.e., finances, hygiene, family support, neighbourhood, etc.)?
**6. What particular situations in your clients' homes highlight risks or challenges for medication management?**
**7. Please describe for me how you approach medication management with your homecare clients and their family members/caregivers?**
8. **What do you think are realistic expectations for your clients and their family members when it comes to medication management at home?**
**9. What factors within your home care organization/work environment either support or hinder your ability to help your clients manage their medications?**
