# Supplementary material for: Seniors managing multiple medications: using mixed methods to view the home care safety lens
Source: BMC Health Serv Res. 2015 Dec 12;15:548. doi: 10.1186/s12913-015-1193-5 (PMC4677040; doi:10.1186/s12913-015-1193-5)
Supplement: Additional file 3: — Table of further examples of vulnerabilities. (PDF 204 kb) [file 12913_2015_1193_MOESM3_ESM.pdf]

**Seniors Managing Multiple Medications: The Home Care Safety Lens**

| Patterns | Additional examples for each pattern |
|---|---|
| **1. Each household harbor's a variety of vulnerabilities that impact their ability to safely manage medication regimens** | **Assumptions:** the wealth of the household which has little bearing on a caregiver and clients ability to manage medication s safely or not. In one household the provider shared "They're quite wealthy. They live in a mansion so they have access to a lot. They have a private home keeper. I think if they needed any extra care put in place, they would definitely be able to sort that out". During an individual interview the caregiver in this home shared her concerns about managing in general due to her increasing forgetfulness and tiredness, as well as a reluctance to ask family or homecare for additional help. The healthcare providers were unaware that she was taking her own medication for early onset dementia.<br><br>**Assumption:** healthcare provider shared "They're a very, very well informed family. They're a very supportive family. They are financially well – relatively well-to-do. So yeah, all that stuff facilitates them – I mean, they're on top of everything where she's concerned"<br><br>**Assumption:** a son was deemed capable of giving the clients intramuscular injections because he was a veterinarian and "familiar with giving injections"<br><br>**Assumption:** An elderly wife was deemed capable of managing her spouse's peritoneal dialysis as the healthcare provider stated "Oh no, they're pretty intelligent …She worked in the health field in some way; I think she was an RPN. So she pretty well understands what I'm talking about and he is into technology so he understands the machine probably better than I do". In fact, the wife shared in her interview that she was never a health care provider at all. This particular caregiver had learned by default or by observing providers, was resourceful in her home environment and had learned to use medical terminology with ease.<br><br>**Storage:** morphine elixir was stored in an unlocked kitchen cupboard. Grandchildren often visited and one one of the researcher visits were observed climbing up to the cupboard to retrieve the morphine for the caregiver to administer to the client. When researchers asked if they ever considered child safety locks the caregiver explained "They'll just climb on the counter to get it to be helpful… So you girls know not to touch that ever" . These examples illustrate that the vulnerabilities related to medication management present safety risks not only for clients and caregivers but also to their visitors. |

| | |
|---|---|
| **2. Every household works hard to sustain adequate supports for their care, including medication issues** | **Relying on family members to assist with taking medications:** One NS participant explained "the doctor gave me these pills and they have to be cut in half and they're so hard. I can't do it myself so my daughter-in-law or my son break them in two for me" .<br><br>**Caregiver burnout:** Comments from caregivers and providers coalesced around issues with getting sufficient home care assistance, inadequate access to respite care, and other chronic issues related to timely access to other team members such as family physician or specialty care. |
| **3. There are varying degrees of sharing accountability for engaging in care between household** | Providers not going over medications lists or medications in the home due to a lack of charting in homes<br><br>A Quebec client reported that hospital personnel rarely checked to ensure that he went home with his medications when discharged, observing that "… the patient doesn't know until they arrive home and find that, "Oh I forgot my meds."<br><br>Ontario nurses indicated that clients refused to answer questions about medications at times, complaining that the paperwork necessary for medication reconciliation took too long to complete.<br><br>In Ontario, providers noted that clients often arrived home from hospital stays with checklists indicating the client had been taught about new medications, including how to do injections, whereas the client and family would state they had not been taught.<br><br>Clients who were managing their own medications could receive new prescriptions that home care remained unaware of for lengthy periods of time. As one provider reported: "We have a client that has been on home care forever and he went into emergency and I found out six months later that he was in emergency and that he had a dose of one of his medications changed. I had no idea". |
| **4. Households put a variety of systems of variable effectiveness into place to manage meds** | Typed medication lists or photocopiers to copy handwritten medication lists to keep organized and have updated lists available.<br>One client explained " Any medication that I purchased, I typed them so I could find them right away"; and a caregiver shared "Dad manages his own medication, he's very good he keeps an updated list everywhere we go he pulls that list out and I've taken to making a few photocopies so when we go to |

| | |
|---|---|
| | see a new division or wherever we go we can leave them with a copy because often he doesn't get his list back".<br><br>Using a food tray to separate medications before filling a dossette<br><br>Takeing medications out of a bubble pac and placing on a china plate<br><br>Storing dropped or omitted medications in spare bottles/jars<br><br>Writing instructions on medication bottles as reminders to clients i.e. breakfast |
| **5. Navigating the healthcare system to obtain an optimum medication regimen requires poly-literacy (health, medication and the healthcare system** | A client who was re-admitted to hospital with a worsening infection because he did not realize the computer printout he received on discharge was actually an antibiotic prescription. |
| **6. There are several systemic challenges to maintaining medication safety in the home** | Providers worried about making a mistake when copying medications from sheet to sheet, and suggested it would be much safer if pharmacies initiated sending forms and electronic copies to family physicians.<br><br>Home medication forms mandated by QC provincial law were frequently not updated.<br><br>Delays with new medication orders showing up in their clients' electronic medical record meant providers could not always rely on this documentation system when trying to do medication reconciliation after transitions from hospital, emergency, physician office or clinic visits.<br><br>In Quebec, providers noted that the medication list was sent to the family physician for verification of prescribed medications was often not returned back to the home.<br><br>Nova Scotia providers sent medication updates to clients' physicians every 3 to 4 months to verify the prescribed regimen, but as one nurse framed it, "Sometimes I wonder if it's even read".<br><br>One Alberta provider noted, full re-assessments were "officially" due every year and as needed in |

| | |
|---|---|
| | between, but when clients came in and out of hospital every month, "…. we couldn't keep up".<br><br>Home support workers in Alberta, Quebec, and Ontario noted they and providers were not always advised of medication updates in a timely fashion by family members, physicians, or partner agencies.<br><br>Sometimes, confusing communication related to divergent messages from different providers. As one client shared, he stopped going to the diabetic clinic because "…. they tell you to do one thing and the doctor tells you to do something else" .<br><br>Providers reported often "feeling a step behind" in keeping up with changes to medications, with many examples related to Coumadin dosages when a client's INR was changing and physicians adjusted the prescribed dose<br><br>Providers expressed frustration with the lack of feedback they received when they noted and relayed concerns regarding clients' medications or medication related issues.<br><br>Providers voiced a desire for more opportunity to debrief and learn from incidents that were being reported.<br><br>Alberta and Quebec providers and clients described scenarios where deficient continuity of care was resolved through involvement of a consistent nurse practitioner, clinic liaison nurse, or other allied health liaison.<br><br>Quebec and Alberta providers also noted that where present, emergency department liaisons with home care were valuable improvements to continuity, recommending more use of these specialized personnel to strengthen the safety of medication management in the home. |
